# Reliability of the Capacity to Consent to Treatment Instrument in Metastatic Cancer

**DOI:** 10.1093/geroni/igaa057.482

**Published:** 2020-12-16

**Authors:** Mackenzie Fowler, Dario Marotta, Richard Kennedy, Kristen Triebel

**Affiliations:** 1 University of Alabama at Birmingham, Birmingham, Alabama, United States; 2 Student, Birmingham, Alabama, United States

## Abstract

Metastatic cancer patients undergo numerous treatment strategies with known cognitive side effects. It is unclear how medical decision-making capacity (MDC) is impacted by cognitive deficits in metastatic cancer. This study examines reliability of an objective measure of MDC compared with self-report. Participants with newly diagnosed metastasis to the brain and other sites were enrolled at the University of Alabama at Birmingham over seven years. At the study visit, participants completed a comprehensive neuropsychological battery including memory and executive functioning, and the Capacity to Consent to Treatment Instrument (CCTI) assessing medico-legal domains of MDC. The CCTI is a reliable and valid instrument containing two vignettes of hypothetical medical situations assessing four standards of consent. We examined reliability between the CCTI and self-reported MDC using Gwet’s AC1 statistic. Participants with brain metastasis with impairment on the CCTI demonstrated significantly lower executive functioning and memory skills than those without impairment (Trails B Raw: 158.20±86.82 vs. 118.90±77.98 p: 0.0248; Digit-Span Raw Forward: 9.13±2.30 vs. 10.12±2.03 p: 0.0254). There were no significant differences between intact and impaired participants with other metastases. Low reliability was observed between self-report and all medico-legal standards on the CCTI across both metastatic groups [Gwet’s AC1 Appreciation: 0.70 (0.58, 0.81); Reasoning: 0.34 (0.16, 0.52); Understanding: 0.44, (0.27, 0.60)]. Self-rating of MDC is unreliable in metastatic cancer patients. Patients with metastases (particularly brain metastases) may lack awareness of deficits in their MDC, so providers must affirm proper autonomy in their decisions.

